# Decision Fatigue in Emergency Medicine: An Exploration of Its Validity

**DOI:** 10.7759/cureus.51267

**Published:** 2023-12-29

**Authors:** Sankalp Yadav, Gautam Rawal, Madhan Jeyaraman

**Affiliations:** 1 Medicine, Shri Madan Lal Khurana Chest Clinic, New Delhi, IND; 2 Respiratory Medical Critical Care, Max Super Speciality Hospital, New Delhi, IND; 3 Orthopaedics, ACS Medical College and Hospital, Dr. MGR Educational and Research Institute, Chennai, IND

**Keywords:** physician well-being, emergency physicians, ego depletion, stress & burnout, decision fatigue

## Abstract

Emergency physicians face a relentless stream of complex, high-stakes decisions in a fast-paced and dynamic environment. The concept of decision fatigue, a phenomenon characterized by a decline in the quality of decision-making after a long sequence of choices, has garnered increasing attention within healthcare. Several investigations show that the number and complexity of decisions made during prolonged shifts correlate with increased self-reported fatigue; however, the effect on clinical decision quality is uncertain. Conversely, a subset of studies found no clear relationship between decision fatigue and errors in clinical judgment. Importantly, some researchers argue that decision fatigue may be mitigated by factors such as experience, training, and support systems. This narrative review highlights the existing literature on decision fatigue among emergency physicians and explores whether this concept holds as a valid concern or remains a myth in the context of their practice.

## Introduction and background

Decision fatigue is a concept that has attracted increasing attention within healthcare, particularly in the context of emergency medicine. Emergency physicians are at the forefront of medical care, making rapid, high-stakes decisions in an environment where lives hang in the balance [1]. The very nature of their work necessitates a continuous series of decisions, from diagnostic choices to treatment plans, often under conditions of extreme stress [2]. The potential presence of decision fatigue among emergency physicians has significant implications for both physician well-being and patient care [3]. This article aims to explore the validity of decision fatigue in the practice of emergency medicine.

## Review

Overview of decision fatigue

Definition and Concept

Decision fatigue is a psychological phenomenon that refers to the deterioration in the quality of decisions made by an individual after a long period of decision-making [4]. It is rooted in the idea that, as humans make choices and decisions throughout the day, the mental and emotional resources required for their decision-making become depleted. Consequently, as these resources diminish, individuals are more likely to make impulsive or less-considered decisions or even avoid making decisions altogether [5].

In the context of healthcare, decision fatigue is particularly relevant to professionals who are required to make numerous decisions, often under time constraints and high levels of stress. Physicians, including emergency physicians, exemplify this concept, as they are routinely faced with complex, critical decisions that can have life-altering consequences for patients. The cognitive demands of diagnosing, treating, and providing care for individuals in emergent situations place these healthcare professionals at risk for decision fatigue [3].

Historical Context

The concept of decision fatigue can be traced back to the early 20th century. Psychologist Walter Lippmann, in his 1922 book *Public Opinion*, discussed the notion of "mental fatigue" and how it could affect decision-making processes [6]. However, it was not until the late 20th century that research in psychology and behavioral economics began formalizing the concept [4].

One key historical milestone was the work of Roy F. Baumeister (2022), who, along with his colleagues, explored the idea of "ego depletion." Ego depletion refers to the idea that self-control and willpower draw upon a finite resource, and as this resource is expended, self-control and decision-making abilities diminish. Baumeister's research, particularly his study on the effects of decision-making on subsequent self-control, laid the foundation for understanding decision fatigue in a broader sense [7].

In healthcare, the historical context of decision fatigue can be tied to the evolving demands of medical practice, especially in high-stress settings like emergency medicine. The proliferation of diagnostic and treatment options, coupled with the need for rapid decisions, has heightened the significance of understanding and addressing decision fatigue among healthcare professionals [3].

Relevance in Healthcare

Decision fatigue is highly relevant in healthcare because of the critical nature of decisions made by healthcare professionals and the potential consequences of suboptimal decision-making. In the context of emergency medicine, where timely and accurate decisions are imperative, the relevance of decision fatigue becomes even more pronounced [3,8].

The healthcare environment, generally, is fraught with numerous decisions: diagnostic choices, treatment plans, medication selection, and prioritization of patient care. Healthcare providers, including emergency physicians, operate under demanding circumstances that can lead to the depletion of cognitive resources. The stress of work, long hours, and constant need for decision-making can contribute to the onset of decision fatigue [8,9].

**Figure 1:**
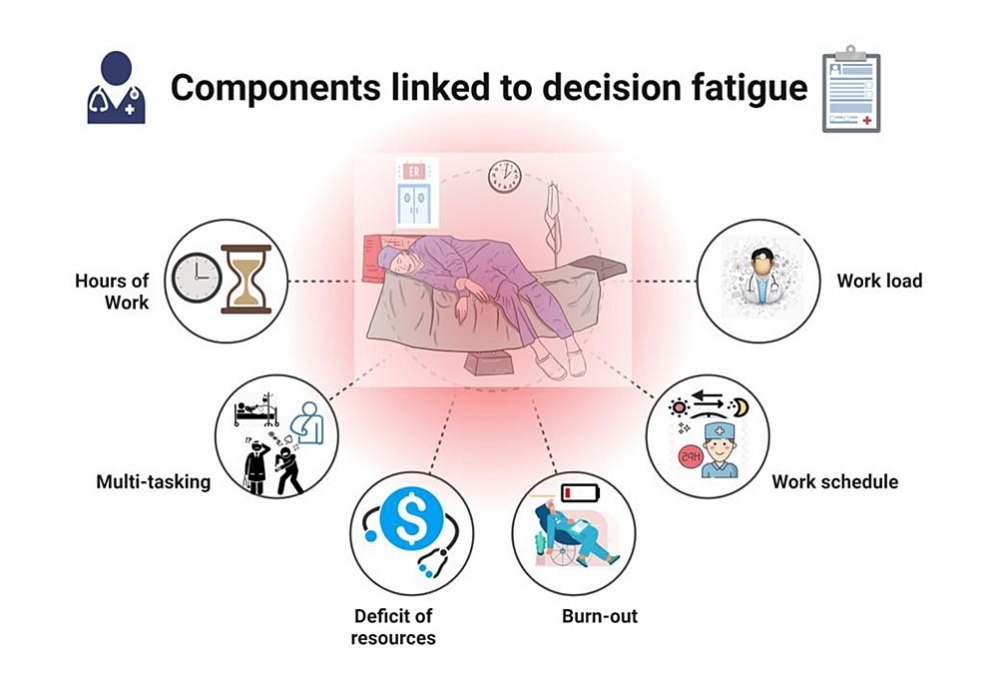
Components of decision fatigue Picture courtesy of Dr. Sankalp Yadav

Moreover, the concept of decision fatigue has implications for patient safety and quality of care. Errors in clinical judgment, medication errors, and suboptimal treatment choices can result from decision fatigue, potentially leading to adverse patient outcomes [4,9]. Recognizing and addressing decision fatigue is crucial for enhancing the well-being of healthcare professionals and, by extension, ensuring the best possible care for patients (Figure 1).

Decision-making in emergency medicine

Complexity of Decisions

Emergency medicine is a field that places a unique set of demands on healthcare providers. It is characterized by the need for rapid and often life-saving decisions. Emergency physicians must assess patients with a wide range of symptoms, illnesses, and injuries often with limited information and in high-stress situations. The complexity of the decisions they face cannot be overstated [10-12].

Diverse presentations: Patients in the emergency department present with a diverse array of medical conditions, from minor or nonspecific complaints to life-threatening emergencies. This diversity demands a wide range of diagnostic and treatment decisions [13,14].

Diagnostic challenges: Diagnosing patients in emergency settings can be particularly challenging. Physicians must make decisions with incomplete information, and misdiagnoses or delays in diagnosis can have serious consequences. One of the specialties with the greatest prevalence of diagnostic errors is emergency medicine [15,16].

Treatment decisions: Once a diagnosis is established, treatment decisions must be made promptly. These decisions range from prescribing medications to performing life-saving procedures [9].

Resource allocation: Emergency departments often face resource constraints, including limited staff, equipment, and beds. Decision-making extends to resource allocation, prioritizing patients based on their medical needs and the available resources [8].

High-Stress Environment

The emergency department is known for its high-stress environment [17]. Physicians work under significant pressure because of factors such as time constraints, uncertainty, and the emotional toll of dealing with critically ill or injured patients. This environment amplifies the cognitive and emotional demands on emergency physicians [17,18].

Time sensitivity: Time is of the essence in emergency medicine. Delays in diagnosis or treatment can have severe consequences, necessitating rapid decision-making [19].

Uncertainty: Physicians often lack complete information when making decisions in the emergency department. This uncertainty adds to the mental workload and can increase the risk of cognitive fatigue [20,21].

Emotional impact: Emergency physicians frequently encounter distressing situations, including traumatic injuries, life-threatening illnesses, and patient deaths. Coping with the emotional toll of these experiences can further deplete cognitive resources [22].

Long shifts: Many emergency physicians work long and irregular shifts, which can lead to physical and mental fatigue, potentially compounding decision fatigue [3,9].

Evidence of decision fatigue among emergency physicians

Self-Reported Fatigue

One avenue for exploring decision fatigue among emergency physicians is through self-reported fatigue. Healthcare providers, including emergency physicians, often experience high levels of stress and exhaustion because of the nature of their work. The subjective experience of fatigue can offer insights into the mental and emotional strain associated with decision-making in this demanding environment [3,23].

Surveys and questionnaires: Researchers have employed surveys and questionnaires to assess self-reported fatigue among emergency physicians. These tools often inquire about the frequency and intensity of decision-related stress and exhaustion. Responses provide valuable qualitative data regarding the perceived burden of making critical decisions [24,25].

Challenges in self-reporting: While self-reporting is a valuable method, it is not without limitations. Physicians may underreport their feelings of fatigue because of professional and social pressures. Moreover, self-reported fatigue does not necessarily correlate directly with decision quality, making it important to consider it in conjunction with other forms of evidence [9,26].

Studies Showing Correlations

Several studies and reviews have reported correlations between decision fatigue and clinical outcomes or decision quality in emergency medicine. These investigations provide quantitative data that suggests potential links between the mental strain associated with decision-making and its impact on healthcare providers and patients [27-33].

Correlation with error rates: Some studies have explored the relationship between decision fatigue and error rates in clinical practice. They investigate whether physicians who experience higher levels of decision fatigue are more prone to making mistakes in diagnosis, treatment, or patient management [9,34].

Assessing decision quality: Other research focuses on the quality of decisions made by emergency physicians. This includes evaluating whether decisions made during extended shifts or under high-stress conditions differ in terms of their appropriateness and effectiveness [35].

Methodological Challenges

Studying decision fatigue among emergency physicians is not without methodological challenges. It is a complex phenomenon influenced by various factors, and designing studies that can accurately capture its presence and impact presents several hurdles [36].

Measurement validity: Ensuring that the tools and methodologies used to assess decision fatigue are valid and reliable is crucial. Variability in measurement instruments can impact the consistency and comparability of results [37].

Confounding variables: The healthcare environment is multifaceted, with numerous variables that can influence decision-making and fatigue. Identifying and controlling for these confounding variables is challenging but essential for accurate findings [37].

Ethical considerations: Conducting research on decision fatigue in emergency medicine must take into account the ethical implications of subjecting physicians to potentially fatiguing conditions. Balancing the need for scientific inquiry with the well-being of healthcare providers is a critical consideration.

Impact on clinical outcomes

Error Rates and Patient Safety

The potential impact of decision fatigue on clinical outcomes, particularly error rates and patient safety, is a topic of paramount concern in emergency medicine.

Increased error rates: Decision fatigue may lead to a higher incidence of errors in clinical practice. Fatigued healthcare providers may make mistakes in diagnosis, medication administration, or patient management. Such errors can compromise patient safety and result in adverse events [38].

Medication errors: In high-stress environments, medication errors are of particular concern. Fatigued physicians might administer the wrong medication, the incorrect dosage, or misinterpret drug interactions, all of which can have severe consequences for patient well-being [3,38].

Delayed diagnoses: Decision fatigue can impair diagnostic accuracy, potentially leading to delayed diagnoses or misdiagnoses. In conditions where early intervention is critical, such delays can lead to adverse patient outcomes [19].

Adverse events: Errors stemming from decision fatigue can contribute to a range of adverse events, including patient harm, increased hospital stays, and, in the worst cases, mortality. The burden of these events extends to healthcare systems and providers, affecting the quality of care and patient trust [27,28].

Clinical Decision Quality

In addition to error rates, the quality of clinical decisions is a central concern when assessing the impact of decision fatigue in emergency medicine.

Inferior decision-making: Decision fatigue may lead to suboptimal clinical decisions. These decisions may not align with evidence-based guidelines or best practices, and they may fail to address the unique needs of individual patients [3,9].

Risk-benefit balance: Fatigued healthcare providers may struggle to weigh the risks and benefits of various treatment options effectively. Inadequate consideration of these factors can lead to subpar clinical outcomes [27,28].

Informed consent: The quality of decision-making also extends to the informed consent process. Patients have a right to understand the potential risks and benefits of treatments, and decision fatigue may compromise the thoroughness of this communication.

Long-Term Implications

The long-term implications of decision fatigue in emergency medicine extend beyond the immediate impact on patient safety and clinical decision quality.

Physician burnout: Chronic exposure to decision fatigue can contribute to physician burnout, with an incidence of 25-78% [3]. The cumulative stress of high-stakes decision-making in a high-stress environment may result in emotional exhaustion, depersonalization, and reduced personal accomplishment [26].

Job satisfaction: Reduced job satisfaction is a concern, as it can lead to decreased morale among emergency physicians. This, in turn, may result in higher turnover rates and recruitment challenges, impacting the stability of healthcare teams [8].

Healthcare costs: Errors and suboptimal clinical decisions driven by decision fatigue can lead to increased healthcare costs. The financial burden of medical interventions to address adverse events, as well as extended hospital stays, can strain healthcare budgets [39,40].

Patient trust: Patient trust is a cornerstone of effective healthcare delivery. When clinical decisions are compromised by decision fatigue, patient trust may erode, affecting the patient-provider relationship and patient compliance with treatment plans [41].

Debates and conflicting evidence

Studies Suggesting No Clear Link

While numerous studies have investigated the concept of decision fatigue among emergency physicians, there remains a body of research that suggests no clear link between decision fatigue and clinical outcomes. These studies have generated debates within the medical community, raising questions about the extent and significance of decision fatigue in emergency medicine [9].

Methodological limitations: Some researchers argue that the existing studies suffer from methodological limitations that may have masked the true impact of decision fatigue. They assert that better-designed investigations are needed to reveal the subtler effects of cognitive fatigue on clinical decisions [4].

Heterogeneity of physician responses: The heterogeneity of physician responses to decision fatigue is another point of contention. Individual differences in resilience, coping strategies, and other factors may mask the overall impact of decision fatigue in group studies [9,42,43].

The Role of Experience and Expertise

The role of experience and expertise in mitigating the effects of decision fatigue is a significant point of debate. Experienced emergency physicians may be better equipped to handle decision-making under high-stress conditions, potentially reducing the impact of decision fatigue [44].

Expertise as a buffer: Seasoned emergency physicians may develop heuristics and streamlined decision-making processes, allowing them to make effective decisions with less cognitive burden. Their accumulated knowledge and experience could serve as a buffer against decision fatigue [44].

Variability in resilience: The resilience of physicians to decision fatigue can vary widely based on individual characteristics and personal coping strategies. Therefore, the impact of experience on decision fatigue may be context-specific and highly individualized.

The Need for Comprehensive Investigations

Debates persist regarding the need for more comprehensive investigations into decision fatigue in emergency medicine. While individual studies have contributed to the understanding of this phenomenon, there is a call for larger, more rigorous, and multifaceted investigations [9].

Longitudinal studies: Longitudinal studies that follow emergency physicians over extended periods could provide valuable insights into how decision fatigue may accumulate and impact clinical practice over time.

Multimodal assessments: Comprehensive investigations may encompass various methods for assessing decision fatigue, including cognitive testing, physiological measurements, and self-reporting. A multi-dimensional approach may reveal nuances that single-mode assessments cannot capture.

Mitigation and coping strategies

Training and Education

Mitigating decision fatigue through training and education is an area of growing interest. Training programs can equip healthcare providers, including emergency physicians, with the knowledge and skills needed to make more effective decisions in high-stress environments [3].

Clinical decision support: Providing emergency physicians with decision support tools and training in their use can aid in navigating complex cases and reducing cognitive fatigue [3,8].

Resilience training: Programs that focus on stress management, emotional regulation, and resilience-building can help physicians cope with the emotional toll of their work and reduce the impact of decision fatigue [45].

Decision Support Tools

The implementation of decision support tools in the clinical setting is gaining traction as a strategy to mitigate decision fatigue. These tools can provide evidence-based recommendations and guidelines, reducing the cognitive load on healthcare providers [3,8,44].

Clinical pathways: Developing standardized clinical pathways can guide decision-making in specific scenarios, helping emergency physicians make consistent and appropriate choices [39,46].

Alert systems: Utilizing alert systems for critical situations, such as sepsis or medication interactions, can prompt timely decisions, reducing the cognitive burden of detecting these issues [47].

Shifting Toward Shared Decision-Making

Shared decision-making is a strategy that involves patients in the decision-making process, particularly in cases where multiple treatment options are available. This approach can alleviate the cognitive burden on physicians and empower patients to participate actively in their care [39].

Informed consent: Engaging patients in discussions about treatment options, risks, and benefits promotes informed consent, improving the patient experience, and reducing the cognitive burden on physicians.

Patient education: Providing patients with educational materials and resources to make informed decisions can streamline the decision-making process and enhance patient-centered care [48].

Implications for emergency medicine practice

Physician Well-Being

The implications of decision fatigue for physician well-being are profound. Acknowledging and addressing decision fatigue can enhance the mental health and job satisfaction of emergency physicians, reducing burnout and turnover rates [3].

Patient-Centered Care

Mitigating decision fatigue can lead to more patient-centered care [48]. Improved decision-making can enhance patient safety and the quality of care provided in emergency medicine, fostering trust and confidence in healthcare services.

Future Directions for Research and Practice

Future research should explore decision fatigue comprehensively, encompassing both its presence and its impact on emergency medicine practice. This will involve investigating individual, organizational, and support system factors, refining mitigation strategies, and promoting a holistic approach to decision-making in high-stress healthcare settings. Addressing decision fatigue is essential for optimizing both the well-being of healthcare providers and the care delivered to patients.

The existence of decision fatigue among emergency physicians remains a subject of debate. While there is evidence of increased self-reported fatigue with the accumulation of decisions during extended shifts, its direct impact on clinical decision quality and patient safety is not definitively established. The complex and multifactorial nature of decision-making in the emergency department demands a nuanced examination of the role of decision fatigue. Further research should aim to disentangle the interplay of variables, including individual resilience, organizational support, and the nature of decisions encountered, to determine the true extent of decision fatigue in this critical clinical context.

## Conclusions

In conclusion, decision fatigue is an intriguing concept within emergency medicine, with implications for physician well-being and patient care. This article provides a snapshot of the current state of the literature, emphasizing the need for more comprehensive investigations to definitively determine whether decision fatigue is a reality or remains a myth among emergency physicians. Addressing this question is essential for optimizing both the work environment and the quality of care delivered in emergency departments.
